# Autoimmune encephalitis with mGluR5 antibodies: A case series from China and review of the literature

**DOI:** 10.3389/fimmu.2023.1146536

**Published:** 2023-03-21

**Authors:** Kundian Guo, Xu Liu, Xue Gong, Aiqing Li, Yue Liu, Xingjie Li, Dong Zhou, Zhen Hong

**Affiliations:** ^1^ Department of Neurology, West China Hospital, Sichuan University, Chengdu, Sichuan, China; ^2^ Institute of Brain Science and Brain-inspired Technology of West China Hospital, Sichuan University, Chengdu, Sichuan, China; ^3^ Department of Neurology, Chengdu Shangjin Nanfu Hospital, Chengdu, Sichuan, China

**Keywords:** autoimmune encephalitis, metabotropic glutamate receptor 5, Ophelia syndrome, China, outcome

## Abstract

**Background:**

Only 15 patients of autoimmune encephalitis with metabotropic glutamate receptor 5 (mGluR5) antibodies have been reported worldwide since 2011, mostly from western countries. Patients with different genetic backgrounds are necessary to further clarify the clinical phenotype and prognosis of this rare disease.

**Objective:**

We initially describe a case series from China to confirm the previous findings, expand the clinical phenotype, and identify the prognostic factors of autoimmune encephalitis with mGluR5 antibodies.

**Methods:**

Observational data with follow-up were prospectively collected from autoimmune encephalitis patients with mGluR5 antibodies. Clinical information and outcomes on current and previously reported cases were combined and analyzed.

**Results:**

We identified five patients (median age 35 years); two were female. The main clinical manifestations were behavioral/personality changes (five of five, 100%) and cognitive disorders (four of five, 80%), accompanied with other neurologic symptoms. Hypoventilation occurred in two (40%) patients, which was life-threatening. One patient had meningoencephalitis, suggesting a new phenotype in anti-mGluR5 encephalitis. All patients received immunotherapy. At the last follow-up (median 18 months), two (40%) patients showed complete recovery, two (40%) patients showed partial recovery, and one (20%) patient died. One (20%) patient had multiple relapses. Together with the 15 previously reported cases, associated tumors occurred in seven of 12 (58%) Western patients vs. one of eight (13%) Chinese patients. Modified Rankin Scale (mRS) scores at the last follow-up (median 31 months) were available in 16 patients. Patients with bad outcomes (mRS > 2, n = 4) were more likely to have hypoventilation at onset and higher mRS scores at peak of the disease.

**Conclusions:**

In patients with different genetic background, as Chinese, the clinical phenotype of anti-mGluR5 encephalitis is similar. Fewer paraneoplastic cases were observed in Chinese patients. Most patients showed good responses to immunotherapy and cancer treatment. The clinical outcomes were favorable in most patients.

## Introduction

Metabotropic glutamate receptors (mGluR) are G-protein coupled receptors activated by the binding of glutamate, the main excitatory neurotransmitter of the nervous system (1). Eight subtypes of mGluR (mGluR1-8) have been cloned and classified into three groups based on their molecular, pharmacological, and signaling properties (1). Group I mGluRs, including mGluR1 and mGluR5, are implicated in diverse processes such as learning, memory, epilepsy, and pain (2). In the past two decades, mGluR1 (3), mGluR5 (4), and mGluR2 (5) have been identified as targets of antibodies in autoimmune neurologic disorders of different characterization. Autoimmune encephalitis with mGluR5 antibodies was first reported in two patients with limbic encephalitis and Hodgkin’s lymphoma (Ophelia syndrome) in 2011 (4). A recent study in an animal model has confirmed the pathogenicity of mGluR5 antibodies, which causes a reduction of mGluR5 clusters in neurons (6). However, as far as we know, since 2011, only 15 autoimmune encephalitis patients with mGluR5 antibodies from 8 studies have been reported worldwide, including three case reports from China (4, 7–13). Given the rarity of anti-mGluR5 encephalitis, additional studies in patients with different genetic backgrounds are necessary to further clarify the clinical spectrum and the disease prognosis.

To confirm the previous findings, expand the clinical phenotype of anti-mGluR5 encephalitis, and report the neurologic outcome, we initially describe a case series of five newly identified patients with anti-mGluR5 encephalitis from China in the current study. We also review the previously reported cases in the literature along with the current data and identify the prognostic factors of anti-mGluR5 encephalitis for the first time.

## Methods

### Study design and identification of patients

This single-center observational study was registered (registration number: ChiCTR1800019762) on the World Health Organization international clinical trial registry platform. Between June 2019 and January 2022, cerebrospinal fluid (CSF) and sera from 2995 patients with suspected autoimmune encephalitis from the Department of Neurology, West China Hospital were investigated with cell-based assay (CBA; Shaanxi MYBiotech Co., Ltd.) for antibodies to mGluR5 with techniques described in the next section. All samples were taken before immunotherapy. These samples were also screened for other neuronal targets [NMDAR, CASPR2, LGI1, AMPAR1, AMPAR2, and GABA_B_R (Euroimmun, Lübeck, Germany); GABA_A_Rα1, GABA_A_Rγ2, GABA_A_Rβ3, GlyRα1, DPPX, IgLON5, mGluR1, dopamin2 receptor, and neurexin-3α (Shaanxi MYBiotech Co., Ltd.)] with CBA and for onconeural antibodies (Hu, Yo, Ri, Ma2, CV2/CRMP5, amphiphysin, Tr, SOX1, Titin, Zic4, Recoverin, and GAD65) with immunoblot analysis (Euroimmun, Lübeck, Germany). Patients were prospectively recruited in the series once the following criteria were met: (1) acute or subacute onset (rapid progression of fewer than three months) of at least one of the following symptoms: behavioral or personality changes, cognitive deficits, sleep disturbances, seizures, decreased level of consciousness, and movement disorders; (2) positive mGluR5 antibodies testing in serum and/or CSF; and (3) reasonable exclusion of other neurological or systemic diseases (7).

Clinical information, including prodromal symptoms, clinical manifestations at the acute phase, auxiliary examinations results (CSF analyses, electroencephalogram [EEG], and brain magnetic resonance imaging [MRI]), tumor association, treatment strategies, and treatment responses were prospectively obtained from the medical record system and standardized questionnaires completed by experienced neurologists (Z.H. and D.Z.) after face-to-face interviews. Follow-up information, including neurologic relapse, residual symptoms, and clinical outcomes, were obtained from regular follow-up at the neurology clinic in West China Hospital. The modified Rankin Scale (mRS) was used to assess symptom severity at peak of the disease and each follow-up, and clinical outcome at the last follow-up.

Outcomes were classified as lack of improvement, partial recovery (if patients had significant improvement but were not back to the baseline condition), complete recovery (if patients returned to the baseline condition), or death (7). Patients with an mRS score ≤ 2 at the last follow-up were also considered to have a good outcome, otherwise to have a bad outcome (14). Relapse was defined as the new onset or worsening of neurologic symptoms after at least two months of the initial improvement or stabilization (14).

### mGluR5 antibodies CBA

To identify mGluR5 antibodies in new patients, human mGluR5 was cloned into pcDNA3.1 vectors. HEK293 cells were used to transfect with the pcDNA3.1 vectors. After 24 hours of transfection, the cells were then fixed with 4% paraformaldehyde for five minutes, washed in phosphate-buffered saline (PBS)-0.1% Tween 20, and ready for antibody detection. Serum with dilution at 1:10 in 0.4% PBS Triton X-100 and CSF without dilution was utilized to incubate cells for one hour at room temperature. Cells were then washed in PBS-0.1% Tween 20 for three times, and incubated at room temperature for 30 minutes with FITC-goat anti-human immunoglobulin G (IgG) (Cat#109-095-170, Jackson ImmunoReaserch, PA, USA), washed again in PBS-0.1% Tween 20, and then determined the reactivity by comparing with HEK293 cells transfected with empty vector plasmids and incubated with the samples from the same patient, using immunofluorescence microscopy by two investigators independently. The antibody titers were determined using serial dilutions (from 1:10 to 1:1000) of patient’s serum and CSF once the samples were positive. The titers were defined as the highest dilution for which the specific fluorescence of HEK293 cells was still visible. The IgG subclasses of patient’s antibodies were also determined in two newly identified patients with mGluR5 antibodies using anti-subclass antibodies specific for IgG1, IgG2, IgG3, and IgG4.

### Review of previously reported autoimmune encephalitis cases with mGluR5 antibodies

We searched PubMed and web of science for all research published in English between October 2011 and December 2022, using the search terms [(mGluR5 OR metabotropic glutamate receptor 5 OR anti-mGluR5 OR anti-metabotropic glutamate receptor 5 OR mGluR5-antibody) and (encephalitis OR autoimmune encephalitis OR Ophelia syndrome)] to identify the previously reported autoimmune encephalitis cases with mGluR5 antibodies in human. To analyze the clinical features, auxiliary examinations results, tumor association, and treatment response, and assess factors potentially associated with the clinical outcomes, current data were reviewed along with the previously reported cases with anti-mGluR5 encephalitis when the following information was available: (1) clinical features at onset and severity (the mRS scores) at the peak of disease; (2) CSF and/or MRI results (including IgG subclasses information available in nine patients); and (3) treatment strategy, clinical outcome, and the mRS scores at the last follow-up.

### Standard protocol approvals and patient consents

This study was approved by the Research Ethics Committee of the Medical School of Sichuan University. Informed consent was obtained from each patient for using their medical records and samples. All data in the study were strictly anonymous.

### Statistical analyses

Statistical analyses and figures were performed with SPSS version 25.0 (IBM Corp., Armonk, NY), GraphPad Prism 8, and R software version 3.6.0. Continuous variables were analyzed using the Mann-Whitney *U* test and shown as medians (interquartile range [IQR]), while categorical variables were analyzed using Fisher’s exact test and shown as frequencies (proportions). Variables potentially associated with the clinical outcomes were compared between patients with bad outcomes vs. good outcomes to identify the prognostic factors. Odds ratios (OR) and medians (using the Hodges-Lehmann method (15, 16)) were used to estimate the difference between the two groups. The corresponding 95% confidence intervals (CIs) of the rate difference and median difference were reported. Two-sided *p*-values of <0.05 were regarded as statistically significant. Given the limited cases of patients and the exploratory nature of the study, multivariate analyses were not performed.

## Results

### Clinical features, results from auxiliary examinations, treatment, and clinical outcomes

Five new patients (patient 1 to patient 5) with mGluR5 antibodies were enrolled. Demographic data, detailed clinical features, auxiliary examination findings, antibody results, treatment strategy, and clinical outcomes are shown in **Table 1**.

**Table 1:** Clinical information, antibody results, and clinical outcomes of five autoimmune encephalitis patients with mGluR5 antibodies.

Patient No., sex, age (years)	Prodromal symptoms	Tumor	Main clinical features; (mRS score at peak of the disease)	CSF analysis	Brain MRI findings; EEG findings	Time from onset to the initial immunotherapy (days)	Treatment	Last follow-up from onset (months); outcome; (mRS score at last follow-up)	mGluR5 Ab; Ab subclass (tested sample)	Additional Ab (tested sample)
1, M, 32	Headache, fever	None	Acute onset personality changes, behavioral changes with irritability, aggressive behavior, apathy, auditory hallucination, memory deficits, attention deficits (MoCA N.A.), difficulties falling asleep; (3). Relapse at 3 month (mRS score 3) and at 28 month (mRS score 3).	<5WBC, increased IgG index, OCB N.A.	At onset and at first relapse: Normal, at second relapse: T2/FLAIR hyperintensities in bilateral hippocampi; Normal at all episodes	24	IVMP, IVIg, followed by tapering oral prednisone; MMF after the second relapse	42; partial recovery, stable after the second relapse, mild residual memory deficits and attention deficits; (1)	CSF: 1:10, S: 1:320; IgG1, 2 (S and CSF)	None
2, M, 36	Headache, flu-like symptoms	None	Acute onset personality changes, behavioral changes with irritability, mania, visual hallucination, difficulties falling asleep, visual deficits; (2)	80 WBC, increased IgG index, OCB neg	T2/FLAIR hyperintensities in unilateral (right) mesiotemporal lobe, cerebral peduncle, thalamus, and putamen; Normal	73	IVMP, IVIg, followed by tapering oral prednisone	24; complete recovery; (0)	CSF: 1:10, S: neg; IgG subclass N.A.	Recoverin (S)
3, F, 35	Headache	Mature teratoma	Acute onset spatial disorientation, prosopagnosia, memory deficits (MoCA N.A.), visual hallucination, generalized seizures, refractory status epilepticus, then rapidly progressive decreased level of consciousness and being in a coma, dystonia, hypoventilation; (5)	120 WBC, increased IgG index, OCB N.A.	T2/FLAIR hyperintensities in bilateral hippocampi; Focal slowing and epileptiform discharge	12	IVMP, IVIg, followed by oral prednisone, surgical removal of the teratoma	6; death; (6)	CSF: 1:100, S: 1:10; IgG subclass N.A.	NMDAR (CSF and S); AMPAR1 and AMPAR2 (CSF)
4, F, 35	Fever, flu-like symptoms	None	Acute onset personality changes, depressed mood, apathy, decreased verbal output, auditory hallucination, memory deficits, executive dysfunction (MoCA 21/30), difficulties falling asleep; (3)	<5WBC, normal IgG index, OCB N.A.	Normal; Normal	4	IVMP, followed by tapering oral prednisone	18; complete recovery; (0)	CSF: neg, S: 1:10; IgG subclass N.A.	None
5, M, 59	Diarrhea, flu-like symptoms	None	Acute onset personality changes, behavioral changes with irritability, aggressive behavior, visual hallucination, aphasia, memory deficits (MoCA N.A.), decreased level of consciousness, meningeal irritation, hypoventilation; (5)	<5WBC, normal IgG index, OCB neg	Diffuse dura mater enhancement on contrast-enhanced T1-weighted imaging; Diffuse slowing	14	IVMP	11; partial recovery, residual cognitive disorders, unable to walk unassisted; (4)	CSF: 1:10, S: 1:100; IgG 1 (S and CSF)	None

Ab, antibody; CSF, cerebrospinal fluid; EEG, electroencephalogram; FLAIR, fluid-attenuated inversion recovery; IgG, immunoglobulin G; IVIg, IV immunoglobulins; IVMP, IV methylprednisolone; mGluR5, metabotropic glutamate receptor 5; MMF, Mycophenolate mofetil; MoCA, Montreal Cognitive Assessment; mRS, Modified Rankin Scale; neg, negative; N.A., not available; OCB, oligoclonal bands; S, serum; WBC, white blood cells/mm3.

The median age at onset was 35 years (range: 32-59 years). Two (40%) patients were female. All patients had prodromal symptoms, including headache, flu-like symptoms, fever, and diarrhea. All patients had behavioral/personality changes. Other encephalitic symptoms included cognitive deficits (four of five, 80%), sleep disturbances (three of five, 60%), decreased level of consciousness (two of five, 40%), movement disorders (one of five, 20%), and generalized seizures (one of five, 20%), manifesting as status epilepticus. Infrequent neurologic symptoms included aphasia, meningitis, prosopagnosia, and visual deficits. Hypoventilation occurred in two (40%) patients, and both needed intensive care at the acute phase. All patients underwent tumor screening, including serum markers and whole-body PET-CT. An associated tumor was found in one (20%) patient, patient 3 (also positive for NMDAR antibodies in serum and CSF), who had a mature ovarian teratoma. The comorbid autoimmune disorder was found in one (20%) patient, patient 1, who had autoimmune hepatitis. The median mRS score was 3 at peak of the disease.

**Figure 1:**
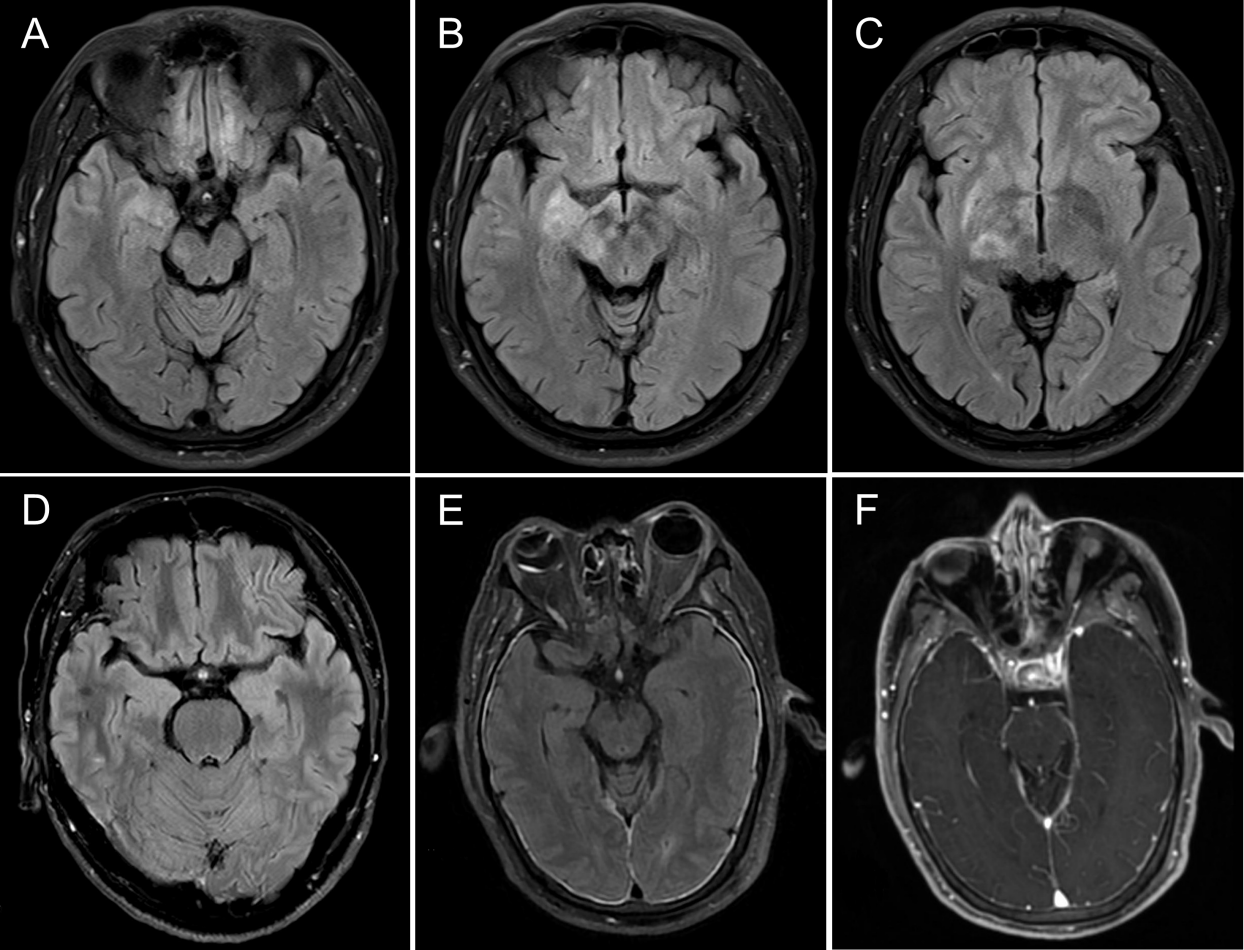
MRI of representative patients with anti-mGluR5 encephalitis in the current study. **(A-C)** Brain MRI of patient 2. Initial brain MRI at disease onset showed T2/fluid-attenuated inversion recovery hyperintensities of the right mesiotemporal lobe **(A)**, cerebral peduncle **(B)**, thalamus, and putamen **(C)**. **(D)** Brain MRI of patient 1. Brain MRI at disease onset and at the first relapse was normal, but at the second relapse, brain MRI showed T2/fluid-attenuated inversion recovery hyperintensity of the bilateral hippocampi. **(E, F)** Brain MRI of patient 5. Initial brain MRI at disease onset showed diffuse T2/fluid-attenuated inversion recovery hyperintensity of the meninges and enhanced on post-gadolinium T1-weighted images.

CSF abnormalities in the routine examination were observed in three (60%) patients. Three (60%) patients showed increased IgG index and two (40%) of them also showed pleocytosis. EEG revealed abnormalities in two (40%) patients. Epileptiform discharge was found in patient 3, who had status epilepticus. A diffuse slowing was observed in patient 5. Specific brain MRI abnormalities occurred in four (80%) patients, including three patients with T2/fluid-attenuated inversion recovery (FLAIR) hyperintensities involving limbic regions and extra-limbic regions, including the thalamus, brainstem, basal ganglia, and cerebellum, and one patient (patient 5) with meningeal enhancement on post-gadolinium T1-weighted imaging. To be mentioned, patient 1, who had a normal brain MRI at the onset and first relapse, found T2/FLAIR hyperintensities in bilateral hippocampi at the second relapse (28 months after the disease onset). Brain MRI of representative patients is shown in **Figure 1**. Brain fluorodeoxyglucose-PET was performed in patient 2, which revealed decreased metabolism of the occipital cortex.

**Figure 2:**
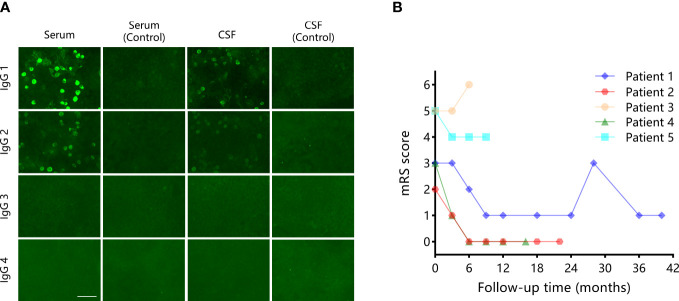
A representative mGluR5 antibody testing result and the mRS scores evaluated over the follow-up of the five patients in the current study. **(A)** mGluR5 IgG subclass results in patient 1. mGluR5 antibodies subclasses were measured on fixed HEK293 cells using a cell-based assay against the mGluR5 expressed at the cell surface. IgG1 and IgG2 were found in the samples. Negative controls: HEK293 cells expressing empty vector plasmids were incubated with the samples from the same patient. Scale bar: 75 μm. **(B)** The mRS scores evaluated over the follow-up of five patients in the current study.

Paired samples were available from all patients. Among these samples, antibodies to mGluR5 were found both in serum and CSF in three (60%), one (20%) only in serum, and one (20%) only in CSF. IgG subclass information was available in two patients. IgG1 was found independently in patient 5 and accompanied by IgG2 in patient 1. **Figure 2A** shows the antibody test result of patient 1. Additional antibodies were found in two (40%) patients, including Recoverin in patient 2 and NMDAR+AMPAR in patient 3.

All patients received first-line immunotherapy (intravenous methylprednisolone, intravenous immunoglobulin, oral prednisone), and one (20%) of them also received second-line immunotherapy (mycophenolate mofetil [MMF]) at relapse. The median time from onset to the initial immunotherapy was 14 days (range: 4-73 days). Patient 3 also had a surgical removal of the tumor. **Figure 2B** shows the mRS scores evaluated over the follow-up of the five patients. At the last follow-up (median 18 months), two (40%) patients showed complete recovery, two (40%) patients showed partial recovery, and one (20%) patient died in the intensive care unit after the withdrawal of the ventilator. The median mRS score at the last follow-up was 1. Repeated tumor screening per year or at relapse during the follow-up did not find newly developed tumors. Patient 1 had multiple relapses at three months and 28 months after the disease onset, respectively. Immunotherapy was still effective in all episodes in this patient.

### Review of the literature

We reviewed the clinical information of 15 previously reported cases from eight studies (4, 7–13) and combined them with that of the five patients in the current study. Detailed information is summarized in **Table 2**.

**Table 2:** Clinical features of 20 autoimmune encephalitis patients with mGluR5 antibodies.

	No. of patients	%
Total ^a^	20	100
Demographics		
Median age at onset (IQR, years)	35.5 (27.5-51.5)	–
Female	9	45
Comorbidity		
Autoimmune disorders	2	10
Tumors	8	40
Hodgkin’s disease	6	30
Other tumors	2	10
Prodromal symptoms	16	80
Headache	10	50
Fever	8	40
Flu-like symptoms	7	35
Weight loss	4	20
Nausea	2	10
Erythema/rash	2	10
Diarrhea	1	5
Neurologic symptoms		
Behavioral or personality changes	17	85
Irritability/mood changes	12	60
Psychosis/hallucination	14	70
Cognitive deficits	15	75
Memory	12	60
Executive	2	10
Attention	3	15
Spatial orientation	3	15
Sleep disturbances	10	50
Seizures	10	50
Generalized seizures	6	30
Focal seizures	1	5
FBDS	1	5
Unknow ^b^	2	10
Status epilepticus	4	20
Decreased level of consciousness	8	40
Movement disorders	6	30
Dystonia	3	15
Tremor	2	10
Myoclonus	1	5
Orofacial dyskinesias	1	5
Ataxia	1	5
Other symptoms		
Hypoventilation	3	15
Aphasia	3	15
Prosopagnosia	2	10
Meningitis	2	10
Visual deficits	1	5
Facial paralysis	1	5
Ophthalmoplegia	1	5
CSF analyses		
CSF abnormalities	17	85
CSF pleocytosis	15	75
CSF OCB (+) or increased IgG index	11/14	79
mGluR5 antibodies testing results		
Positive in CSF	14/17	82
Positive in serum	15/16	94
Positive in both CSF and serum (paired samples)	9/13	69
Additional autoantibodies in samples	5	25
Abnormal EEG	10/19	53
Epileptiform discharge	5/19	26
Diffuse slowing	4/19	21
Myogenic artifact	1/19	5
Brain MRI at onset or relapse		
T2/FLAIR hyperintensities or meningeal enhancement	12	60
Abnormalities in limbic regions	5	25
Abnormalities in extra-limbic regions	9	45
Treatment		
Immunotherapy	17	85
First-line immunotherapy	17	85
IVMP (corticosteroids)	17	85
IVIg	8	40
PE	3	15
Second-line immunotherapy	4	20
RTX	2	10
MMF	2	10
Cancer treatment	8/8	100
Intensive care required	6	30
No treatment	1	5
Outcome at the last follow-up		
Median time from onset to last follow-up (IQR, months)	20 (6-48)	–
Complete recovery	10	50
Partial recovery	9	45
Lack of improvement	0	0
Death	1	5
Relapse	3	15

EEG, Electroencephalogram; FLAIR, fluid-attenuated inversion recovery; IgG, immunoglobulin G; IQR, interquartile range; IVIG, IV immunoglobulins; IVMP, IV methylprednisolone; mGluR5, metabotropic glutamate receptor 5; MMF, mycophenolate mofetil; OCB, oligoclonal bands, PE, plasma exchange; RTX, rituximab.

a Five from the current study and 15 from previous cases (4, 7–13).

b One case was reminiscent of psychogenic nonepileptic seizures but reported improvement under levetiracetam and worsening after discontinuation; one case only mentioned status epilepticus (7, 9).

**Figure 3:**
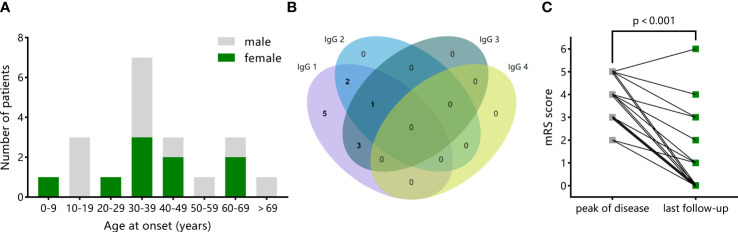
Demographics, mGluR5 antibodies subclass distribution, and clinical outcomes in all patients with anti-mGluR5 encephalitis. **(A)** The distribution of patients by age and sex in anti-mGluR5 encephalitis. **(B)** Venn diagram of the distribution of IgG subclasses in 11 patients with available information. IgG1 was found in all patients independently (5) or accompanied by IgG2 (2), IgG3 (3), or both IgG2 and IgG3 (1). **(C)** Comparison of mRS scores at peak of the disease with that at the last follow-up in 16 patients with available information.

The median age at onset was 35.5 years (IQR 27.5-51.5 years); 45% of patients were female. The distribution of age at the onset and sex is shown in **Figure 3A**. Prodromal symptoms were common, which occurred in 16 of 20 (80%) patients. The most frequent neurologic symptoms were behavioral/personality changes (17 of 20, 85%), followed by cognitive deficits (15 of 20, 75%), sleep disturbances (10 of 20, 50%), seizures (10 of 20, 50%, including status epilepticus in two children and two adults), decreased level of consciousness (eight of 20, 40%), and movement disorders (six of 20, 30%). Hypoventilation occurred in three (15%) patients. Six (30%) patients needed intensive care at the acute phase. The median mRS score was 4 at peak of the disease.

Associated tumors were found in eight (40%) patients, including six with Hodgkin’s disease, one with small cell lung cancer, and one with mature ovarian teratoma (who also positive for NMDAR antibodies in serum and CSF). The tumor association had no significant difference between males and females (*p* = 0.288). We also noticed that there were seven of 12 (58%) paraneoplastic cases in Western patients vs. one of eight (13%) paraneoplastic cases in Chinese patients (six of eight Chinese patients underwent whole-body PET-CT for tumor screening), with a difference showing the tendency toward statistical significance (*p* = 0.07).

CSF abnormalities were observed in 17 (85%) patients. Fifteen (75%) patients showed pleocytosis [median 31, range 6-396 white blood cells/mm^3^] and 11 of 14 (79%) patients had the oligoclonal band or increased IgG index. EEG revealed abnormalities in 10 of 19 (53%) patients, including five patients with epileptiform discharge (two children and three adults, all had seizures), four patients with diffuse slowing (three children and one adult), and one with myogenic artifact associated with a faciobrachial dystonic seizure. Specific brain MRI abnormalities at onset or relapse occurred in 12 (60%) patients. Brain fluorodeoxyglucose-PET in four patients (two with normal brain MRI) all demonstrated decreased metabolism involving the temporoparietal cortex, occipital cortex, or cerebellum.

All patients had positive results to mGluR5 antibodies in CSF and/or serum. Among 13 patients with paired samples, antibodies to mGluR5 were found both in serum and CSF in nine (69%) patients. IgG subclass information was available in 11 patients, shown in **Figure 3B**. Additional antibodies were found in five (25%) patients, including SOX1, Recoverin, LGI1, NMDAR+AMPAR, and NMDAR+MOG, respectively.

Seventeen (85%) patients received first-line immunotherapy and four of them also received second-line immunotherapy. All patients with associated tumors received chemotherapy, radiotherapy, or surgical removal as cancer treatment. At the last follow-up (median 20 months), 10 (50%) patients showed complete recovery, nine (45%) patients showed partial recovery, and one (5%) patient died. Neurologic relapse occurred in three (15%) patients. The median time from onset to the first relapse was 16 months (range 3-30 months). Immunotherapy (and cancer treatment) was still effective at all episodes among patients with relapses.

The mRS scores information at the last follow-up (median 31 months) was available in 16 patients, including 12 (75%) with good outcomes and four (25%) with bad outcomes (**Table 3**). The median mRS score at the last follow-up was 1, significantly decreasing from the median mRS score of 4 at peak of the disease (**Figure 3C**, p<0.001). Compared to patients with good outcomes, patients with bad outcomes had higher frequency of hypoventilation (75% vs. 0; OR 58.3, 95% CI 1.92, 1771; *p* = 0.02) and higher severity at peak of the disease, reflected by higher mRS score (median 5 vs. 4; diff median 1, 95% CI 0, 2; *p* = 0.042). There were no significant differences in the demographic features, symptoms other than hypoventilation, comorbid tumors, CSF testing results, brain MRI abnormalities, and immunotherapy strategy between the two groups.

**Table 3:** Comparison of autoimmune encephalitis patients with mGluR5 antibodies according to good or bad outcome.

	Good outcome	Bad outcome	OR; diff medians^b^	95% CI^b^
Patients (total 16), n (%)^a^	12 (75)	4 (25)		
Demographics				
Median age at onset (IQR, years)	33.5 (19-37)	47.5 (27.75-63.75)	19.5	-24, 44
Female, n (%)	5/12 (42)	2/4 (50)	1.40	0.14, 13.6
Comorbidity				
Tumor, n (%)	5/12 (42)	2/4 (50)	1.40	0.14, 13.6
Prodromal symptoms, n (%)	10/12 (83)	4/4 (100)	2.14	0.08, 54.2
Neurologic symptoms, n (%)				
Behavioral or personality changes	12/12 (100)	3/4 (75)	0.09	0.003, 2.83
Cognitive deficits	10/12 (83)	4/4 (100)	2.14	0.08, 54.2
Sleep disturbances	9/12 (75)	1/4 (25)	0.44	0.04, 5.01
Seizures	5/12 (42)	2/4 (50)	1.40	0.14, 13.6
Status epilepticus	1/12 (8)	2/4 (50)	11.0	0.65, 187
Decreased level of consciousness	5/12 (42)	3/4 (75)	4.20	0.33, 53.1
Movement disorders	3/12 (25)	3/4 (75)	9.00	0.66, 123
Other symptoms, n (%)				
Hypoventilation	0/12 (0)	3/4 (75)	58.3	**1.92, 1771**
CSF analyses, n (%)				
CSF pleocytosis	10/12 (83)	3/4 (75)	0.60	0.04, 9.16
CSF OCB (+) or increased IgG index	8/9 (89)	2/4 (50)	0.13	0.007, 2.18
Brain MRI at onset or relapse, n (%)				
T2/FLAIR hyperintensities or meningeal enhancement	5/12 (42)	4/4 (100)	12.3	0.54, 279
mGluR5 antibodies results, n (%)				
Positive in both CSF and serum (paired samples)	5/7 (71)	3/3 (100)	3.18	0.12, 87.9
Additional autoantibodies in samples, n (%)	1/12 (8)	2/4 (50)	11.0	0.65, 187
Immunotherapy, n (%)				
First-line immunotherapy (IVMP, IVIg, PE, oral prednisone), n (%)	9/12 (75)	4/4 (100)	3.32	0.14, 78.8
Second-line immunotherapy (RTX, MMF, among others), n (%)	2/12 (17)	1/4 (25)	1.67	0.11, 25.4
Severity (mRS score) at peak of the disease (median)	4	5	1	**0, 2**
Outcome at the last follow-up				
Median time from onset to last follow-up (IQR, months)	40 (20-48)	15 (10-30)	-13.5	-42, 14
Last follow-up mRS score (median)	0	4	3	**2, 5**

FLAIR, fluid-attenuated inversion recovery; IgG, immunoglobulin G; IQR, interquartile range; IVIG, IV immunoglobulins; IVMP, IV methylprednisolone; mGluR5, metabotropic glutamate receptor 5; MMF, mycophenolate mofetil; OCB, oligoclonal bands, PE, plasma exchange; RTX, rituximab.

Bold entries indicate p < 0.05

a Five from the current study and 11 from previously published cases (4, 7–9).

b Based on the Hodges-Lehmann method for median differences (15, 16).

## Discussion

This study described a case series of anti-mGluR5 encephalitis from China, which aim to confirm the previous findings, expand the clinical phenotype of anti-mGluR5 encephalitis, and identify the prognostic factors of clinical outcome.

All except one patient in our case series were in their 30s, which agrees with the previous study (7). Unlike anti-NMDAR encephalitis, that occurs predominantly in females, we observed no gender difference in mGluR5 encephalitis, confirming the previous findings (7, 14). All patients in our case series had prodromal symptoms preceding the neurologic symptoms and developed behavioral/personality changes, mood changes, or psychiatric symptoms, ranging from negative symptoms such as apathy and decreased verbal output to positive symptoms such as behavioral changes with irritability, mania, and hallucination. Besides, cognitive deficit is another prominent symptom in our case series, which occurred in 80% of patients. Most of them had one or multiple cognitive domain impairments, and memory loss was the most common. These results were similar to the previous findings (7). A recent study has revealed that mGluR5 antibodies can reduce the level of mGluR5 in the hippocampus, causing memory loss and anxiety in mice, which was in agreement with the clinical phenotype of anti-mGluR5 encephalitis (6). Other common neurologic symptoms include sleep disturbances, seizures, decreased level of consciousness, and movement disorders, which is align with previous studies (4, 7–9). It is notable that a patient in our case series showed symptoms of both meningitis and encephalitis. Brain MRI revealed diffuse dura mater enhancement on contrast-enhanced T1-weighted imaging. Thorough evaluations for infectious, rheumatic, and malignant etiologies were performed to rule out other differential diagnoses, and no evidence of additional neuronal antibodies was found. Therefore, meningoencephalitis should be considered a new phenotype of anti-mGluR5 encephalitis and expand the clinical spectrum. Interestingly, a recent case report described a patient with mGluR5 antibody-associated Guillain-Barré syndrome without other neuropsychiatric symptoms (not reviewed in this study) (17). Given the extreme rarity of the disease, further studies of future cases should be conducted to clarify if the term “anti-mGluR5 encephalitis” should be replaced by “mGluR5 antibody-associated disease” to define the disease more appropriately.

Although most patients in our case series showed pleocytosis or increased IgG index in CSF analysis, two patients had normal CSF findings in routine examination, as mentioned in some patients with anti-mGluR5 encephalitis in the previous study or other types of autoimmune encephalitis (e.g., mGluR1, NMDAR) (12, 14, 18). Therefore, mGluR5 antibodies should be screened in suspected patients despite having normal results in routine CSF examination. Brain MRI abnormalities at onset or relapse were found in 80% of patients in our case series, which is more frequent than that reported by Spatola et al. (7) As described in the previous study (7), although anti-mGluR5 encephalitis was always considered a form of limbic encephalitis, extra-limbic lesions could also be involved independently or combined with limbic lesions on brain MRI. Notably, patient 1 in our case series, who had a normal brain MRI at disease onset, showed bilateral hippocampi lesions on repeat brain MRI at the second relapse. Therefore, a repeat brain MRI during follow-up is important, especially in patients who are suspected of having a relapse of the disease. Besides, patients in the current study and the previous study who underwent a brain fluorodeoxyglucose-PET all showed decreased metabolism of the cortex or cerebellum, suggesting the potential diagnostic value of brain fluorodeoxyglucose-PET, especially in MRI-negative patients (7).

We noticed that two patients in our case series found additional antibodies in the samples, as observed in many other types of autoimmune encephalitis, such as anti-AMPAR encephalitis and anti-GABA_B_R encephalitis (19, 20). Taking together with the previous studies, five patients with anti-mGluR5 encephalitis had additional antibodies and all showed atypical symptoms: the patient with SOX1 antibodies had progressive ophthalmoplegia (7), the patient with Recoverin antibodies had visual deficits and confirmed retinopathy, the patient with NMDAR+AMPAR antibodies had refractory status epilepticus, the patient with NMDAR+MOG antibodies had cerebral cortical encephalitis (11), and the patient with LGI1 antibodies had faciobrachial dystonic seizures (12). In some of these cases, additional antibodies were pathogenically relevant, resulting a complex clinical pictures (e.g., Recoverin antibodies and retinopathy (21), MOG antibodies and cerebral cortical encephalitis (22), and LGI1 antibodies and faciobrachial dystonic seizures (23)). Similar findings have been reported in other types of autoimmune encephalitis, such as anti-AMPAR encephalitis (20). It is worth mentioning that the clinical implication of coexisting Recoverin antibodies in patient 2, who had found no associated tumor, should be interpreted with caution, as there may be a risk of a false positive test result. Future studies are required to determine which antibody plays the major pathogenic role in patients with multiple antibodies.

Over half of the Western patients with anti-mGluR5 encephalitis had associated tumors (mainly Hodgkin’s disease), however, together with our data, only 13% of Chinese patients had associated tumors, and none of them had Hodgkin’s disease at the last follow-up. The difference in the frequency of paraneoplastic cases between Western patients and Chinese patients closely approaches the statistical significance (*p* = 0.07), most likely due to the limited sample size. One of the possible reasons is the differences in genetic background between different races, as we had reported in our previous studies of anti-NMDAR encephalitis and anti-CASPR2 encephalitis (24, 25). This finding should be confirmed in future cases with longer follow-up since tumors can be occult and diagnosed years after recovery from autoimmune encephalitis in some patients (26). It is worth noting that the only Chinese patient with associated tumor in our case series, who had mature teratoma, found coexisting NMDAR antibodies. It is well-known that NMDAR antibodies had been reported to be associated with teratoma in previous studies. Besides, the patient also had a clinical phenotype of anti-NMDAR encephalitis (27). Therefore, mGluR5 antibodies may not be the major pathogenic ones responsible for the clinical picture in this patient.

In agreement with the previous studies, most patients with anti-mGluR5 encephalitis in our case series showed good responses to the immunotherapy. Combined with the previous data, all except one patient with anti-mGluR5 encephalitis showed complete or partial recovery at the last follow-up. Three-quarters of patients (with available information) had good outcomes at the last follow-up. The mortality was 5% among all cases, close to the clinical outcome observed in anti-NMDAR encephalitis (14, 24). The prognostic factors of bad clinical outcomes were hypoventilation at the episode and higher mRS scores at the peak of the disease. However, no significant difference was detected in the frequency of tumor association between the good outcome group and the bad outcome group, as observed in anti-NMDAR encephalitis (14, 24). Together with the previous cases, relapses were observed in 15% of patients with anti-mGluR5 encephalitis, as reported in anti-NMDAR encephalitis (14, 24). Besides, we first reported a patient with multiple relapses, who had clinical improvement after reinitiating immunotherapy in each relapse as at the first episode. Therefore, the importance of a long-term follow-up in patients with anti-mGluR5 encephalitis should be highlighted, as suggested by Spatola et al. (7) To be mentioned, combined with the previous data, only four patients received second-line immunotherapy, such as RTX at the acute phase or MMF as maintenance therapy. Previous studies in anti-NMDAR and anti-AMPAR encephalitis had revealed the association between lower relapse risk and aggressive treatment (14, 20). This association is awaiting further validation in more extensive prospective cohort studies of anti-mGluR5 encephalitis.

Our study has several limitations. First, given the rarity of this disease and the consequent small sample size, multivariate analyses were not performed to determine the independent prognostic factors of clinical outcome, and the effect of immunotherapy could not be assessed in more detail. Second, some meaningful information was not available from the previous studies, such as the time from onset to immunotherapy and the time from immunotherapy to clinical improvement. Therefore, some factors potentially associated with the clinical outcome could not be analyzed (28). Besides, some patients had relatively short follow-up, which may cause an underestimate of the number of patients with relapses or associated tumors. In addition, the retrospective analysis of the current and previous studies increased the risk of bias, especially when analyzing the response to immunotherapy and the strategy of immunotherapy. Future prospective multi-center studies in larger cohorts with longer follow-ups should be conducted to provide more information about this rare disease. Despite the limitations, we have described five newly identified patients of anti-mGluR5 encephalitis from China, which has provided more evidence to support the previous findings and expanded the clinical spectrum of anti-mGluR5 encephalitis. Besides, we found a lower frequency of paraneoplastic cases in Chinese patients than in Western patients. We also reviewed all reported cases with anti-mGluR5 encephalitis so far and determined the prognostic factors of clinical outcome, which may promote a better understanding of the prognosis of this rare disease.

## Data availability statement

The raw data supporting the conclusions of this article will be made available by the authors, without undue reservation.

## Ethics statement

The studies involving human participants were reviewed and approved by the Ethics Committee of West China Hospital of Sichuan University (Approved No. of ethic committee: 292). The patients/participants provided their written informed consent to participate in this study.

## Author contributions

KG conceptualized and designed the study, collected the data, carried out the statistical analysis, interpreted the data, and drafted the manuscript. XuL, XG, AL, YL, and XiL collected the data. DZ revised the manuscript. ZH conceptualized and designed the study and revised the manuscript. All authors contributed to the article and approved the submitted version.
